# From symptoms to function: the PAD-S decision matrix for severe mental illness—a transdiagnostic clinical translation framework for ICD-11/ICF-aligned psychotherapy documentation

**DOI:** 10.3389/fpsyt.2026.1689702

**Published:** 2026-07-01

**Authors:** Eik Niederlohmann

**Affiliations:** Department of Psychosomatic Medicine and Psychotherapy, Kliniken Erlabrunn, Breitenbrunn, Germany

**Keywords:** benchmarking, clinical decision support, computational psychotherapy, documentation, functional recovery, human-final digital mental health, ICD-11, ICF

## Abstract

Severe mental illness (SMI) is used here as a functional and service-relevant category: mental disorders associated with substantial, persistent, or recurrent restrictions in major life activities, role participation, treatment stability, or safety. Psychotherapy in SMI requires more than symptom reduction. Clinicians must repeatedly decide whether to deepen emotional work, downshift to regulation, address avoidance, protect emerging progress, or intervene against shame-driven self-attack. These decisions are clinically central but often remain poorly documented and difficult to supervise, compare, or validate. This Hypothesis & Theory manuscript proposes an SMI-specific clinical translation of the Perceive-Assess-Dose-Safeguard (PAD-S) decision matrix and the related Conflict-Square Algorithm (CSA) as a sparse, human-final decision language for such moments. PAD-S/CSA organizes session cues into four process nodes: Progression (PRO), Anxiety/Affect tolerance (ANX), Defense/Avoidance (DEF), and Superego/Shame attack (SUP). Each node is linked to tolerance thresholds, calibrated therapist moves, safeguards, and expected functional impact. The framework connects three levels without collapsing them: the International Classification of Diseases, 11th Revision (ICD-11), together with the Clinical Descriptions and Diagnostic Requirements (CDDR), anchors diagnosis and severity context; the International Classification of Functioning, Disability and Health (ICF) and the Mini-ICF-APP rating for activity and participation restrictions in mental disorders anchor activity and participation; PAD-S/CSA documents session-level process decisions. The manuscript clarifies novelty relative to related PAD-S/CSA publications, defines SMI operationally, provides plain-language node definitions, gives an SMI-specific adaptation table, illustrates a worked micro-sequence and two brief vignettes, and proposes a staged validation agenda. Digital extensions are framed as optional annotation, summarization, and research support; clinical responsibility remains human-final.

## 1 Introduction

Psychotherapy for severe mental illness is delivered under conditions of diagnostic heterogeneity, high functional impairment, fluctuating risk, and fragmented service responsibilities. A diagnosis may explain why treatment is needed, but it does not by itself describe what a therapist should do in a particular moment when a patient becomes disorganized, collapses into shame, avoids contact, or shows a small but fragile sign of progress. Conversely, rich clinical formulation often remains narrative and difficult to translate into documentation, supervision, benchmarking, or mental-health informatics (4–10, 16–24).

The present article addresses this gap by proposing a severe mental illness (SMI)-specific clinical translation of the Perceive-Assess-Dose-Safeguard (PAD-S) decision matrix and the related Conflict-Square Algorithm (CSA). PAD-S/CSA is not presented as a replacement for diagnosis, case formulation, treatment modality, or therapist judgment. It is proposed as a sparse process notation for selected micro-decisions in which safety, tolerance, shame, avoidance, and functional recovery determine the next therapeutic move. Its practical question is deliberately simple: what is happening now, how much can be tolerated, what is the safest useful next step, and how should this be documented? (1–3).

The revised manuscript also clarifies the relation to related PAD-S/CSA publications. Because the present manuscript was originally submitted before those articles became formally available, the current version explicitly differentiates its contribution. The general state-action grammar, four-node notation, and shared-representation architecture have been described elsewhere; the new contribution here is the SMI-focused clinical translation that links the session process to International Classification of Diseases, 11th Revision (ICD-11) severity language, International Classification of Functioning, Disability and Health (ICF)/Mini-ICF-APP functioning, human-final documentation, supervision, benchmarking, and implementation in complex care pathways.

### 1.1 Operational definition of severe mental illness in this article

SMI is used as a pragmatic, transdiagnostic, service-relevant category defined primarily by serious functional impairment rather than by one diagnosis. It includes psychosis-spectrum disorders, bipolar disorders, severe persistent or recurrent depressive presentations, complex personality-related presentations, trauma-related disorders, and mixed or comorbid conditions when they are associated with substantial impairment in work or education, self-care, relationships, social participation, treatment stability, risk management, or need for coordinated long-term care. **Table 1** summarizes this operational definition and explains why both psychosis-spectrum and severe depressive vignettes are consistent with the manuscript’s scope (8–10).

**Table 1:** Operational SMI definition used for the PAD-S clinical translation.

Dimension	Operational meaning in this manuscript
Diagnostic scope	Psychosis-spectrum, bipolar, severe depressive, trauma-related, personality-related, and mixed or comorbid conditions.
Functional criterion	Major life activities, work/education, self-care, relationships, social participation, or independent living are substantially restricted.
Clinical risk criterion	Care may involve self-harm risk, psychotic intensification, cognitive-perceptual disruption, shame collapse, relapse risk, or destabilization under interpersonal load.
Service criterion	The patient often requires coordinated, stepped, integrated, or long-term care across psychotherapy, psychiatry, rehabilitation, nursing, and social services.
Why this matters for PAD-S/CSA	The same symptom label can imply different session-level decisions; functional impairment and tolerance determine how direct, activating, stabilizing, or safeguarding the next move should be.

### 1.2 Relation to related PAD-S/CSA publications and novelty of the present article

The three now-published PAD-S/CSA articles create a programmatic background but also require explicit differentiation. **Table 2** differentiates the present SMI-focused manuscript from those related publications. The manuscript should be read neither as a duplicate of those papers nor as a claim that the basic architecture is first introduced here. Its distinct contribution is the clinical, functional, and implementation-oriented translation for SMI care (1–3).

**Table 2:** Relation to related PAD-S/CSA publications and new contribution of the present manuscript.

Related publication	Primary contribution of that publication	New contribution of the present manuscript
PAD-S state-action grammar (1)	Formalizes PAD-S as a safety-gated grammar for psychotherapy micro-decisions and computational psychiatry.	Translates PAD-S into SMI clinical documentation, functional targets, and service-facing supervision logic.
CSA four-node framework (2)	Defines the four-node CSA notation and its relationship to psychotherapy and functional diagnosis.	Explains how the four nodes should be applied conservatively in SMI, where tolerance, CPD, shame, and risk alter the next step.
Shared representation layer (3)	Positions PAD-S/CSA as a candidate representation layer for psychotherapy, digital phenotyping, and simulation.	Keeps the present paper clinically grounded: PAD-S/CSA is used as a human-final documentation and supervision language before any advanced computation.
Present SMI-focused paper	–	Defines SMI operationally; links PAD-S/CSA to ICD-11/CDDR, ICF/Mini-ICF-APP, episode lines, and SMI-specific safeguards; provides a validation roadmap.

### 1.3 Reader roadmap: from complex theory to a usable clinical notation

**Figure 1:**
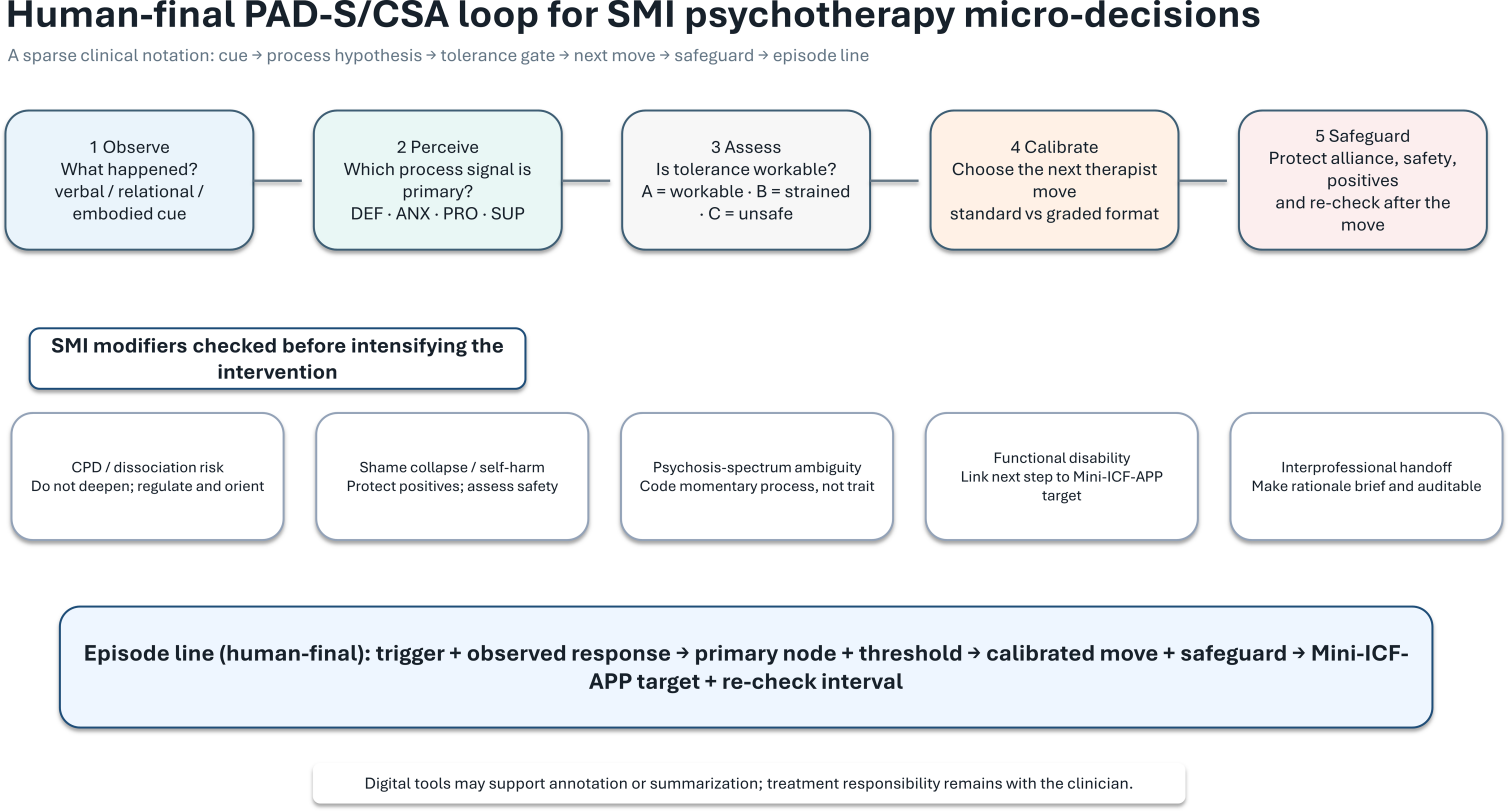
Human-final Perceive-Assess-Dose-Safeguard (PAD-S)/Conflict-Square Algorithm (CSA) loop for severe mental illness (SMI) psychotherapy micro-decisions. The workflow translates session cues into an auditable episode line through five therapist-led steps: observe, perceive, assess, calibrate, and safeguard. The episode line links the observed trigger, patient response, node and threshold, calibrated therapeutic move, safeguard, Mini-ICF-APP target, and recheck interval. The lower strip summarizes SMI-specific modifiers that should be checked before increasing intervention intensity, including cognitive-perceptual disruption (CPD)/dissociation risk, shame collapse or self-harm risk, psychosis-spectrum ambiguity, functional disability, and need for interprofessional handoff. Digital tools may support annotation or summarization; treatment decisions remain human-final.

For readers not trained in Intensive Short-Term Dynamic Psychotherapy (ISTDP), the easiest entry point is not psychodynamic terminology but the practical question: “What is happening now, and what would make the next therapist move safer and more useful?” **Figure 1** provides a self-contained overview of the human-final PAD-S/CSA loop. The important message is that the model does not decide treatment; it helps the clinician make a narrow part of the clinical reasoning explicit and auditable.

In practical terms, **Figure 1** turns an abstract algorithm into a clinical handrail. A therapist does not need to “think computationally” during the session. The therapist notices a cue, forms a process hypothesis, checks tolerance, chooses a calibrated next move, and documents only the decision-relevant layer afterward.

## 2 From symptoms to function: why ICD-11, ICF, and PAD-S are complementary

**Figure 2:**
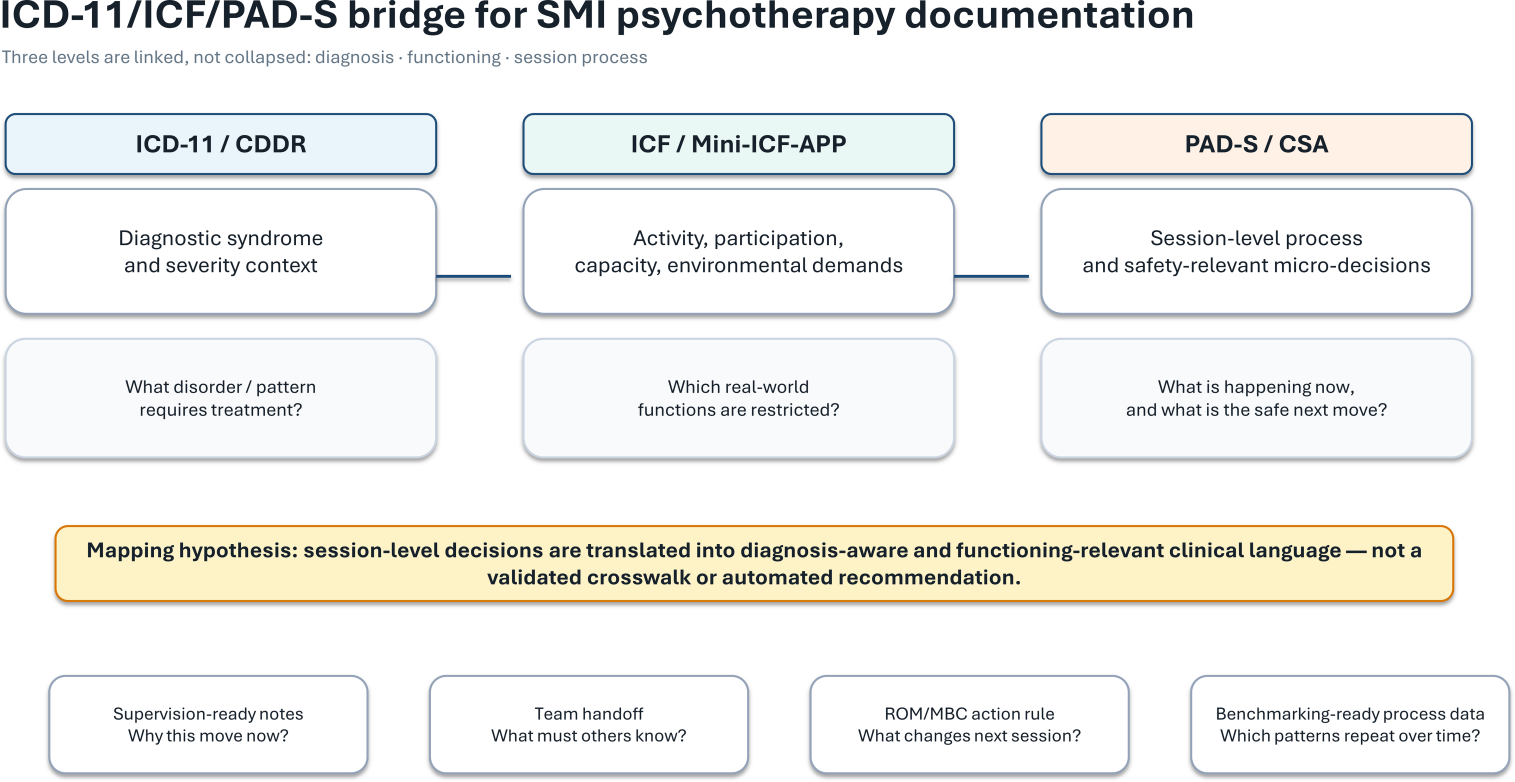
International Classification of Diseases, 11th Revision/International Classification of Functioning, Disability and Health/Perceive-Assess-Dose-Safeguard (ICD-11/ICF/PAD-S) bridge for severe mental illness (SMI) psychotherapy documentation. The figure separates three complementary levels of clinical description: International Classification of Diseases, 11th Revision (ICD-11)/Clinical Descriptions and Diagnostic Requirements (CDDR) diagnostic and severity language, International Classification of Functioning, Disability and Health (ICF)/Mini-ICF-APP functioning and participation assessment, and Perceive-Assess-Dose-Safeguard/Conflict-Square Algorithm (PAD-S/CSA) episode-line notation for session-level psychotherapy process. The bridge is proposed as an implementation-oriented mapping hypothesis, not as a validated ontology, formal crosswalk, or automated treatment recommendation. Intended outputs include supervision-ready notes, team handoffs, routine outcome monitoring/measurement-based care (ROM/MBC) action rules, and benchmarking-ready process data.

ICD-11, ICF/Mini-ICF-APP, and PAD-S/CSA answer different clinical questions. ICD-11/CDDR anchors diagnosis and severity context. ICF and Mini-ICF-APP describe activity, participation, capacity, and environmental demands. PAD-S/CSA adds a session-level process layer: what the therapist observed, how tolerance and safety were estimated, what intervention format was selected, and what functional change was expected. **Figure 2** illustrates this three-level bridge (4–7, 11–15).

The proposed linkage is therefore intentionally modest. PAD-S/CSA does not claim to be a formal ICD-11-ICF ontology or validated crosswalk. It proposes that selected session-level decisions can be translated into diagnosis-aware and functioning-relevant language. This helps clinicians document not only “the patient felt worse” or “the patient improved,” but which process signal led to which calibrated action and which functional target should be rechecked (4–7).

The Mini-ICF-APP is especially useful because it translates psychiatric impairment into capacity domains that can be observed and discussed in treatment planning, rehabilitation, work ability assessment, and social participation. In SMI care, this functional layer is crucial: the clinically relevant question is often not only whether symptoms decrease, but whether the patient can sustain relationships, attend appointments, tolerate groups, manage daily structure, or return to work or education (11–15).

## 3 The PAD-S/CSA nodes in plain clinical language

The four nodes are not patient types and not a complete theory of the mind. They are momentary process hypotheses. A patient may move through several nodes within one session. The therapist’s task is to identify the primary process signal, check the tolerance threshold, and choose the next move. **Table 3** translates the nodes into plain clinical language and highlights why each node was retained (35–46).

**Table 3:** Four node definitions and selection rationale translated into plain clinical language.

Node	Plain-language question	Typical markers	Immediate implication/why retained
DEF: defense/avoidance	Is the patient protecting themselves by moving away from feeling, contact, reality testing, or agency?	Intellectualizing, topic shifts, passivity, blaming, splitting, projective narrative, compliance without contact.	Clarify or challenge only within tolerance; retained because avoidance is observable, action-changing, transdiagnostic, and functionally costly.
ANX: arousal/tolerance	How much emotional or relational load can the patient safely tolerate now?	Somatic anxiety, agitation, shutdown, confusion, derealization, thought blocking, perceptual disruption.	ANX regulates pacing; retained because it is the main stop-rule node in fragile SMI presentations.
PRO: progression	Is there a workable sign of agency, contact, integration, or functional movement?	Affect labeling, curiosity, new action, more coherent narrative, relational flexibility, follow-through.	Consolidate and protect gains; retained because therapy must track functional recovery, not only pathology.
SUP: shame/self-attack	Is progress, closeness, or exposure being attacked by punitive shame or self-hatred?	Global self-criticism, contempt toward self, shame collapse, guilt without repair, self-harm urges after progress.	Protect positives and reduce punitive process before intensifying; retained because shame can convert progress into risk.

### 3.1 Why these four nodes?

The four nodes were selected through theory-informed pragmatic synthesis. They were not derived from a fitted statistical model and should not be interpreted as empirically discovered latent variables. Their psychodynamic lineage is explicit, particularly in Experiential Dynamic Therapy (EDT)/Intensive Short-Term Dynamic Psychotherapy (ISTDP) concepts of defense, anxiety tolerance, affective progression, and punitive self-attack. However, the labels are deliberately translated into broader process language so that clinicians from cognitive-behavioral therapy for psychosis (CBTp), dialectical behavior therapy (DBT), mentalization-based therapy (MBT), acceptance and commitment therapy (ACT), supportive, rehabilitation-oriented, and integrative backgrounds can use the same notation without adopting ISTDP as a treatment model (35–46).

A process coordinate was retained only if it met seven criteria: observable in session, action-changing, safety-relevant, transdiagnostic, orientation-translatable, function-linkable, and codable. This selection logic matters because SMI services require a notation that is clinically meaningful but sparse enough to be taught, documented, audited, and tested.

### 3.2 Thresholds as clinical tolerance gates

The A-C threshold language is a clinical shorthand, not a psychometric scale. Threshold A indicates workable tolerance and coherent collaboration; threshold B indicates rising strain, narrowed reflective capacity, or early instability; and threshold C indicates overload, cognitive-perceptual disruption (CPD), dissociation, disorganization, or safety risk. **Table 4** translates this shorthand into concrete stop-rule implications. For SMI care, this threshold logic is often more important than the node itself: the same DEF, ANX, PRO, or SUP signal may call for very different interventions depending on tolerance (36–41, 45).

**Table 4:** SMI tolerance thresholds and stop-rule implications.

Threshold	Clinical meaning	Recommended PAD-S stance
A: workable	Coherent speech, reflective capacity, stable attention, manageable affect, alliance contact remains available.	Standard format may be used: deepen, clarify, expose, or consolidate while continuing to monitor tolerance.
B: strained	Arousal rises; shame spikes; attention narrows; patient oscillates between contact and avoidance; coherence is still partly available.	Graded format: shorter interventions, more validation, slower tempo, frequent rechecks, explicit permission to pause.
C: unsafe/overloaded	CPD, dissociation, derealization, thought blocking, marked disorganization, self-harm risk, or loss of collaborative contact.	Stop deepening. Downshift to orientation, grounding, stabilization, safety planning, medication/medical review, or interprofessional support as indicated.

## 4 SMI-specific adaptations of the general PAD-S/CSA model

The SMI translation changes the risk calculus of the general model. In lower-risk psychotherapy, a therapist may treat a defensive detour, a rise in anxiety, or a shame signal primarily as an opportunity for deeper work. In SMI, the same signal may indicate that the patient is approaching disorganization, dissociation, self-harm risk, psychotic intensification, or rupture. **Table 5** is therefore the clinical core of this manuscript: it shows how the general PAD-S/CSA architecture must be adapted for SMI care (8–10, 21–29).

**Table 5:** SMI-specific adaptations of the general PAD-S/CSA architecture.

SMI challenge	Why the general model is insufficient without adaptation	SMI-specific adaptation
Fragile affect tolerance	Affect-focused or exposure-based work can rapidly exceed tolerance.	Give ANX/CPD stop rules priority over depth; use shorter exposure windows, grounding, and more frequent rechecks.
Psychosis-spectrum ambiguity	Withdrawal, suspiciousness, flat affect, or unusual content may reflect symptom, medication, trauma, defense, relationship signal, or overload.	Code nodes as momentary hypotheses, not trait labels; keep differential diagnosis, medication, sleep, substances, and context in view.
Shame collapse and self-harm risk	Progress or closeness can trigger punitive self-attack and destabilization.	Treat SUP as a safety node; protect positives, assess risk, slow down, and coordinate supports when needed.
Functional disability	Symptom talk alone may not show whether life capacity is improving.	Attach episode lines to Mini-ICF-APP domains such as endurance, planning, flexibility, contacts, and self-care.
Interprofessional fragmentation	Narrative psychotherapy notes are often hard to use for team handoffs, rehabilitation, or social medicine.	Use concise episode lines that communicate trigger, process, threshold, action, safeguard, functional target, and recheck.
Digital health inequality	SMI patients may vary widely in digital literacy and service access.	Keep PAD-S usable as a low-tech documentation language before adding apps, dashboards, or artificial intelligence (AI) tools.
Artificial intelligence (AI) and documentation risk	Generative systems can introduce errors or overconfident summaries.	Use digital tools only as optional support; require clinician review, consent, privacy safeguards, and crisis escalation procedures.

The practical consequence is conservative: in SMI, “more therapeutic intensity” is not automatically better. PAD-S/CSA encourages an explicit distinction between useful activation and unsafe overload. This is particularly important when the patient appears detached, compliant, ashamed, or confused, because these presentations can be mistaken for lack of motivation rather than limited tolerance (36–41, 45).

## 5 From clinical cue to episode line: a worked example

**Figure 3:**
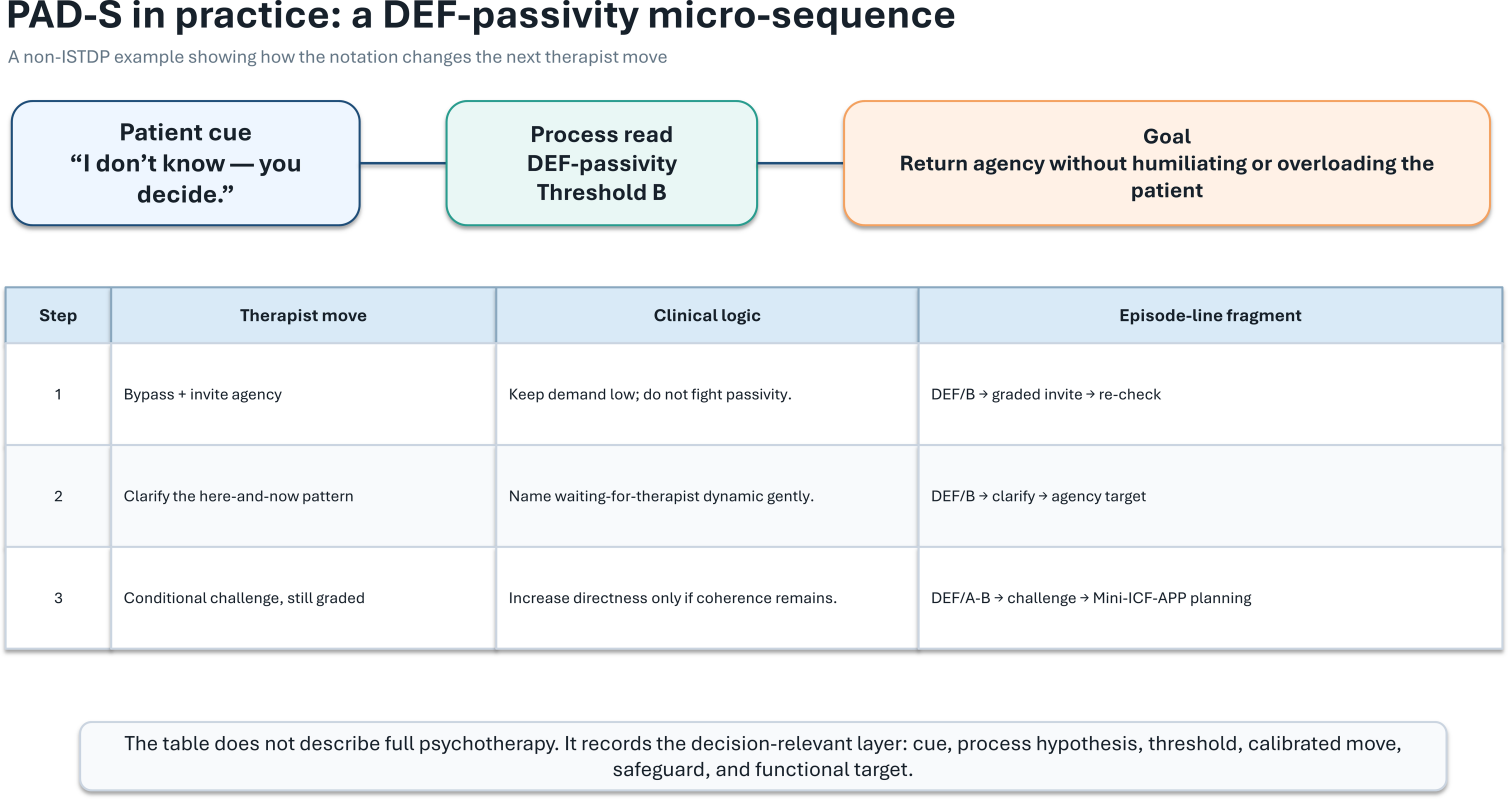
Perceive-Assess-Dose-Safeguard (PAD-S) in practice: a defense/avoidance (DEF)-passivity micro-sequence. Panel A shows how a patient cue is translated into a clinician-adjudicated process hypothesis and threshold estimate. Panel B shows three graded therapist moves: bypassing and inviting agency, clarifying the here-and-now interpersonal pattern, and using a conditional challenge only if coherence remains. The example illustrates documentation logic rather than automated treatment decision-making.

A common barrier for non-ISTDP readers is that terms such as defense, dose, or superego can sound school-specific or abstract. **Figure 3** therefore uses an ordinary clinical moment: a patient gives up agency and asks the therapist to decide. PAD-S/CSA does not prescribe a school-specific technique. It helps the clinician document why the next move should be gentle, graded, and agency-restoring rather than overly directive or confrontational.

This example illustrates the difference between rich psychotherapy and sparse documentation. The therapist still needs formulation, empathy, timing, tone, and alliance sensitivity. PAD-S/CSA documents only the decision-relevant layer: what cue was observed, what process was hypothesized, how tolerance was estimated, what move was chosen, which safeguard was used, and what functional target should be rechecked. **Table 6** provides a minimal template for such episode lines (23–28, 46).

**Table 6:** Minimal PAD-S episode-line schema for routine documentation, supervision, and transcript annotation.

Field	Example from Figure 3	Purpose
Trigger	Patient says: “I don’t know—you decide.”	Defines the observable starting point rather than an interpretation alone.
Patient response/marker	Passivity, waiting for therapist, reduced agency, still coherent.	Keeps the code anchored in visible process.
Primary node + threshold	DEF-passivity, threshold B.	States the process hypothesis and tolerance level.
Calibrated therapist move	Graded invitation to choose one small target; later gentle clarification.	Shows how the intervention was adjusted.
Safeguard	Short statements, no humiliation, recheck coherence and shame.	Documents why the move was safe enough.
Functional target	Mini-ICF-APP planning/structuring: one patient-owned decision before next session.	Links session process to functioning.
Recheck	Next session: did the patient choose or enact one small step?	Makes the episode line testable over time.

## 6 Clinical vignettes

The following composite vignettes are intentionally brief. Their purpose is not to demonstrate full treatment, but to show how PAD-S/CSA changes documentation and supervision. In each vignette, the clinical question is: what is the dominant process signal, what is the tolerance threshold, what is the next safe move, and which functional target should be rechecked?

### 6.1 Psychosis-spectrum presentation with fragile affect tolerance

A 24-year-old patient with episodic psychotic symptoms and marked social withdrawal enters therapy after repeated hospitalizations. At intake, Mini-ICF-APP indicates moderate to severe impairments in endurance, flexibility, and contacts. Early sessions show coherent collaboration for 5-10 min, followed by thought blocking and derealization when interpersonal themes arise. PAD-S/CSA formulation: primary ANX at threshold B-to-C with secondary DEF intellectualization. Next move: downshift to grounding and brief mentalizing, use predictable time limits, and postpone deeper affect work. Functional target: attend one structured group segment with an agreed exit plan and recheck CPD markers afterward (11–15, 21, 42, 45).

### 6.2 Severe persistent depressive presentation with shame collapse and functional disability

A 41-year-old patient with severe persistent depression, complex trauma history, and intermittent self-harm reports pervasive self-disgust and hopelessness. Symptoms fluctuate, but disability is stable: Mini-ICF-APP indicates severe impairment in self-care and occupational capacity. In sessions, emotional activation quickly triggers harsh self-attack and collapse. PAD-S/CSA formulation: primary SUP at threshold B with secondary ANX. Next move: protect the emerging positive, name the punitive process compassionately, slow the affective work, and assess self-harm risk explicitly. Functional target: one small self-care action and one low-intensity contact, reviewed within two sessions (8–15, 19, 20, 39, 40).

The depressive vignette is therefore not a generic depression case. It illustrates the manuscript’s functional SMI definition: severe impairment, risk, chronicity, and need for coordinated care make the case relevant to an SMI framework even without psychotic symptoms (8–10).

## 7 Implementation: documentation, training, and service integration

Implementation in SMI services requires more than a promising model. It requires feasible training, fidelity support, and integration with existing workflows. PAD-S/CSA can be introduced as a minimal common language across therapists, psychiatrists, nurses, social workers, and rehabilitation staff. Its first use does not require advanced digital infrastructure: a clinician can write a one-line episode note in ordinary documentation. More technical forms of transcript annotation, JavaScript Object Notation (JSON)-like schemas, dashboards, process modeling, or large language model (LLM)-assisted summarization should remain supplementary and subject to human review (16–34, 47, 48).

For training, the sequence should be simple: first learn to identify everyday clinical cues, and then name the primary node, estimate threshold, select a calibrated move, and document the safeguard and functional target. This sequence makes PAD-S/CSA usable for non-ISTDP therapists because the entry point is clinical logic rather than school-specific terminology. It also makes the notation usable for computational psychologists because the output has a stable state-action structure without requiring the computational reader to infer the full clinical formulation (19–24, 36–41, 46).

## 8 Research agenda and testable predictions

Because PAD-S/CSA is proposed as a Hypothesis & Theory framework, its value depends on empirical testing. The main claims are not that PAD-S/CSA is already effective but that the notation is feasible, teachable, reliable enough for annotation after training, useful for supervision, and potentially associated with safer calibration and better functional outcomes. **Table 7** summarizes the staged validation roadmap (19–24, 34, 47–51).

**Table 7:** Staged validation roadmap for reliability, feasibility, effectiveness, safety, implementation, and computational process research.

Stage	Primary question	Example design and endpoint
1. Feasibility	Can clinicians use the notation without excessive burden?	Brief training pilot; completion time, usability, perceived clinical relevance.
2. Reliability	Can trained raters agree on node and threshold?	Transcript/vignette annotation study; interrater reliability for primary node and A-C threshold.
3. Clinical safety	Does PAD-S/CSA improve recognition of CPD, shame collapse, and unsafe escalation?	Simulation or supervision study; detection of stop-rule moments and appropriate downshifts.
4. Functional linkage	Do episode lines predict or guide Mini-ICF-APP-relevant functional targets?	Longitudinal pilot; association between episode-line patterns and capacity/participation change.
5. Implementation	Can teams integrate the notation into routine documentation and supervision?	Hybrid effectiveness-implementation study; burden, acceptability, fidelity, sustainability.
6. Computational analysis	Can human-adjudicated episode lines support process modeling without replacing clinicians?	Transcript corpus; supervised annotation, model discovery, error analysis, human-in-the-loop review.

## 9 Discussion

### 9.1 Main contribution

The main contribution of this manuscript is not a new psychotherapy school and not a claim that four nodes capture the whole therapeutic relationship. The contribution is a clinical translation proposal: selected micro-decisions in SMI psychotherapy can be documented in a sparse, safety-aware, human-final format that links clinical process, functioning, supervision, and later validation. This is especially relevant for SMI care, where high-risk moments may be missed when documentation remains either purely narrative or purely symptom-focused (1–7, 11–15, 23–34).

### 9.2 Why this may matter for non-ISTDP and computational readers

For non-ISTDP clinicians, PAD-S/CSA can be understood as a practical pacing language. It asks whether the patient is moving toward function, becoming overloaded, avoiding contact or agency, or attacking themselves through shame. For computational readers, PAD-S/CSA provides a clinically constrained state-action vocabulary. It is intentionally smaller than psychotherapy itself because it is designed to support annotation, supervision, and validation rather than simulate the whole mind or replace the therapist (35–51).

### 9.3 Limitations and claim calibration

Several limitations are central. First, PAD-S/CSA is not yet validated as an intervention, training method, or outcome-improving system. Second, the node definitions are theory-informed and clinically pragmatic, not empirically discovered latent dimensions. Third, PAD-S/CSA does not replace full-case formulation, diagnosis-specific risk management, alliance monitoring, patient preference, cultural formulation, unconscious meaning, transference-countertransference work, or therapist responsiveness. Fourth, the ICD-11/ICF linkage is a mapping hypothesis, not a validated crosswalk. Fifth, digital extensions carry risks of privacy loss, documentation error, bias, overconfidence, and inappropriate automation; therefore, any digital implementation must remain human-final and governed by explicit safety procedures (23–28, 34, 47–51).

## 10 Conclusion

PAD-S/CSA is proposed here as an SMI-specific, ICD-11/ICF-aligned clinical translation framework for documenting, supervising, benchmarking, and eventually validating safety-relevant psychotherapy micro-decisions. Its value lies in making a narrow but consequential layer of clinical reasoning explicit: cue, process hypothesis, threshold, calibrated move, safeguard, functional target, and recheck. The framework should be tested empirically and implemented conservatively. If validated, it could help bridge psychotherapy practice, functional recovery, measurement-based care, and mental-health informatics without replacing therapist judgment or the complexity of the therapeutic relationship (1–7, 11–34).

## Data Availability

The original contributions presented in the study are included in the article/**Supplementary Material**. Further inquiries can be directed to the corresponding author.

## Glossary

AI: Artificial intelligence.
ANX: Anxiety/arousal and affect tolerance: how much emotional or relational load the patient can safely tolerate now.
CBTp: Cognitive-behavioral therapy for psychosis.
CDDR: Clinical Descriptions and Diagnostic Requirements for ICD-11 mental, behavioral and neurodevelopmental disorders.
CPD: Cognitive-perceptual disruption: confusion, derealization, thought blocking, perceptual distortion, or disorganized processing under emotional or interpersonal load.
CSA: Conflict-Square Algorithm: the four-node process notation related to PAD-S.
DEF: Defense/avoidance: protective movement away from feeling, contact, agency, or reality testing.
EDT/ISTDP: Experiential Dynamic Therapy/Intensive Short-Term Dynamic Psychotherapy. These inform some clinical heuristics but are not required for PAD-S/CSA use.
ICD-11: International Classification of Diseases, 11th Revision.
ICF: International Classification of Functioning, Disability and Health.
JSON: JavaScript Object Notation.
LLM: Large language model.
Mini-ICF-APP: Rating of Activity and Participation Restrictions in Mental Disorders.
PAD-S: Perceive-Assess-Dose-Safeguard: a therapist-led loop for micro-decision calibration.
PRO: Progression: observable therapeutic movement toward agency, emotional clarity, relational flexibility, or functional participation.
ROM/MBC: Routine outcome monitoring/measurement-based care.
SMI: Severe mental illness, defined functionally here by substantial impairment, risk, persistence, recurrence, multimorbidity, or need for coordinated care.
SUP: Superego/shame attack: punitive self-attack, shame collapse, or moral threat that reduces agency and safety
