# Supplementary material for: From symptoms to function: the PAD-S decision matrix for severe mental illness—a transdiagnostic clinical translation framework for ICD-11/ICF-aligned psychotherapy documentation
Source: Front Psychiatry. 2026 Jul 1;17:1689702. doi: 10.3389/fpsyt.2026.1689702 (PMC13370903; doi:10.3389/fpsyt.2026.1689702)
Supplement: Supplementary file 1 [file Table1.docx]

**Supplementary Material S1**

*PAD-S/CSA clinician-facing codebook and decision matrix (aligned with the revised manuscript)*

# Purpose and scope

This supplement provides the clinician-facing operational reference for the PAD-S/CSA framework used in the main manuscript. It is intended to make the notation usable for supervision, routine documentation, and future annotation studies. It is not a standalone treatment manual, diagnostic instrument, autonomous algorithm, or substitute for case formulation and risk management.

| **Human-final use:** All labels and episode lines are clinician-adjudicated. Digital tools, if used, may assist with transcription, summarization, or candidate annotation, but treatment decisions and safety judgments remain the responsibility of the clinician. |
| --- |

**Table S1.1. Operating principles for PAD-S/CSA use in SMI care.**

| **Principle** | **Operational meaning for use in SMI psychotherapy** |
| --- | --- |
| Momentary process hypothesis | PRO, ANX, DEF, and SUP are coded as current process signals, not as patient types or fixed traits. |
| Safety before intensity | In SMI, threshold C or B-to-C drift overrides technique: downshift, regulate, protect positives, and reassess risk. |
| Function-first documentation | Each episode line should connect the session event to an expected functional target or capacity domain. |
| Dose means calibration | In PAD-S, dose refers to qualitative intervention directness/intensity, not a metric quantity. |
| Sparse by design | Only the decision-relevant layer is documented: cue, node, threshold, move, safeguard, functional target, re-check. |

# 1. Node quick guide

**Table S1.2. Plain-language node guide for non-ISTDP readers.**

| **Node** | **Plain-language question** | **Typical observable cues** | **First clinical implication** | **Function link** |
| --- | --- | --- | --- | --- |
| PRO Progression | Is the patient moving toward more agency, contact, affect clarity, or functional action? | Names wish/need; regulated affect; coherent narrative; accepts help; tries a new interpersonal or self-care step. | Consolidate and translate progress into small functional action; protect gains from shame backlash. | Participation, contacts, self-care, work/education, decision making. |
| ANX Anxiety / affect tolerance | Is the patient becoming too activated, confused, dissociated, or physiologically overloaded? | Tension; GI flip; nausea; fogging; spacing out; thought blocking; derealization; agitation or shutdown. | Regulate pace. If B-to-C or C appears, stop deepening and orient/ground before any further activation. | Endurance, planning, group integration, mobility/safety, treatment stability. |
| DEF Defense / avoidance | Is the patient avoiding affect, agency, closeness, or reality contact in a way that blocks useful work? | Topic shifting; intellectualizing; debating; blaming; passivity; monologuing; joking away affect; compliance without engagement. | Clarify gently and link avoidance to cost if tolerance allows; use graded format if fragility increases. | Decision making, assertiveness, flexibility, contacts, follow-through. |
| SUP Shame / self-attack | Is the patient attacking themselves or collapsing after contact, praise, progress, or affect? | Harsh self-criticism; global defectiveness; contempt toward self; joy-to-attack-to-collapse; self-harm urges; moral injury themes. | Protect positives, de-shame, assess risk, and postpone deepening when shame escalates. | Self-care, occupational capacity, intimate relations, endurance, safety planning. |

# 2. Threshold quick guide

**Table S1.3. A-C tolerance thresholds and intervention implications.**

| **Threshold** | **Clinical meaning** | **What it looks like** | **Default intervention format** | **Safeguard** |
| --- | --- | --- | --- | --- |
| A Regulated/workable | Affect is symbolizable; collaboration and reflection are available. | Coherent speech; oriented; can pause, reflect, and respond; no CPD or dissociation. | Standard format may be possible: concise clarification, affect focus, exposure, or consolidation. | Monitor for delayed ANX or SUP escalation. |
| B Narrowing | Tolerance is narrowing; reflective capacity is reduced but not lost. | Tension, pressure, rapid speech, detours, micro-shame, early confusion, reduced eye contact. | Graded format: shorter moves, slower pace, smaller exposure windows, frequent check-back. | Use explicit permission to pause; re-check body, orientation, alliance. |
| B-to-C Drift toward overload | The patient may cross into collapse, CPD, dissociation, or shame shutdown if intensity increases. | Fogging, thought blocking, derealization, smooth-muscle anxiety, abrupt shame, shutdown or agitation. | Downshift: stop challenge/deepening, regulate, name safety, protect positives. | Treat as a stop-rule zone, especially in SMI. |
| C Collapse/overload | Therapeutic activation is no longer safe or useful in that moment. | Disorientation, CPD, severe dissociation, psychotic intensification, shame collapse, self-harm risk. | No deepening. Stabilize, ground, orient, safety-plan, coordinate care if needed. | Document risk and follow local clinical protocols. |

# 3. Node by threshold decision matrix

**Table S1.4. Practical Node x Threshold matrix for next-step calibration.**

| **Node** | **A: regulated** | **B: narrowing** | **B-to-C: drift** | **C: collapse/overload** |
| --- | --- | --- | --- | --- |
| PRO | Validate progress; link to one functional action; plan re-check. | Reinforce micro-step; keep exposure small; watch for shame backlash. | Protect positives; pause and check ANX/SUP; reduce demand. | Rebuild safety; no performance pressure; coordinate supports. |
| ANX | Allow brief activation if clinically indicated; track body and cognition. | Use graded exposure, pacing, breath/orientation, and short interventions. | Prioritize regulation; postpone deeper affect work. | Stop, orient, ground, reduce stimulation; consider safety/medical/medication review if indicated. |
| DEF | Clarify avoidance and cost; invite agency or affect in short form. | Partial clarification only; keep focus small; strengthen alliance. | Stop challenge; regulate; return to ANX or SUP safeguards. | No confrontation; co-regulate and re-establish orientation/safety. |
| SUP | Reality-test punitive belief; name cost of self-attack; protect agency. | Protect positives; compassionately name the pattern; slow pace. | Stop-rule: protect positives, de-shame, assess risk. | Shame repair, safety plan, external support, no deepening. |

# 4. Minimal episode-line data dictionary

**Table S1.5. Minimal episode-line fields for routine documentation and annotation.**

| **Field** | **Type** | **Required?** | **Description / example** |
| --- | --- | --- | --- |
| episode_id | string | yes | Unique identifier: session plus sequence, e.g., S03_E07. |
| trigger | short text | yes | Clinician prompt, relational cue, patient event, or situational stressor. |
| observed_response | short text | yes | Verbal, paraverbal, nonverbal, cognitive, affective, or relational cue. |
| primary_node | categorical | yes | PRO, ANX, DEF, or SUP. |
| secondary_node | categorical | optional | At most one secondary node if clinically relevant. |
| threshold | categorical | yes | A, B, B-to-C, or C. |
| calibrated_move | short text | yes | Therapist next move, e.g., clarify, ground, protect positives, validate, conditional challenge. |
| safeguard | short text | yes | Stop rule, grounding, de-shaming, alliance repair, safety plan, team handoff. |
| functional_target | short text | yes | Mini-ICF-APP related target or participation goal. |
| recheck_interval | short text | recommended | Same session, next session, 2-4 sessions, or 6-12 weeks depending on target. |
| annotator_id | string | research only | Rater code for annotation studies. |
| notes | short text | optional | Context that prevents over-interpretation. |

# 5. Optional JSON-like schema for technical teams

| **Use caution:** This schema is included to support reproducible research and transcript annotation. It should not be interpreted as an automated decision system. |
| --- |

{
 "episode_id": "S03_E07",
 "trigger": "Patient asks therapist to decide for them",
 "observed_response": "Passive compliance; low agency; coherent and oriented",
 "primary_node": "DEF",
 "secondary_node": null,
 "threshold": "B",
 "calibrated_move": "graded clarification and invitation of agency",
 "safeguard": "keep focus small; check anxiety and shame",
 "functional_target": "decision making / assertiveness",
 "recheck_interval": "next session",
 "human_final_review": true
}

# 6. What the codebook should not be used for

- It should not be used to label patients as defensive, fragile, or shame-based personalities.
- It should not be used to bypass diagnosis-specific risk management, cultural formulation, informed consent, or local safety procedures.
- It should not be used to automate psychotherapy or recommend treatment without clinician review.
- It should not be interpreted as an empirically validated intervention; it is a proposed documentation and supervision framework requiring validation.
