# Supplementary material for: From symptoms to function: the PAD-S decision matrix for severe mental illness—a transdiagnostic clinical translation framework for ICD-11/ICF-aligned psychotherapy documentation
Source: Front Psychiatry. 2026 Jul 1;17:1689702. doi: 10.3389/fpsyt.2026.1689702 (PMC13370903; doi:10.3389/fpsyt.2026.1689702)
Supplement: Supplementary file 2 [file Table2.docx]

**Supplementary Material S2**

*Annotated micro-sequences and rater training pack (fictionalized composites)*

# Purpose and evidence status

This supplement illustrates how PAD-S/CSA episode lines can be derived from brief clinical dialogue. The examples are fictionalized composites for training and illustration only. They do not represent verbatim patient material and should not be treated as evidence that PAD-S/CSA improves outcomes. The intended research use is rater training, supervision, and future reliability testing.

| **Didactic note:** The examples are deliberately written in plain clinical language so that the logic can be followed without ISTDP training: clinical cue -> process hypothesis -> threshold -> next move -> safeguard -> functional target. |
| --- |

# 1. Coding legend

**Table S2.1. Episode-line coding legend.**

| **Element** | **Meaning for coding** |
| --- | --- |
| Trigger | The therapist prompt, relational event, symptom cue, or external stressor that starts the decision point. |
| Observed response | What the patient says or does, including affect, body signals, cognition, behavior, alliance, or risk signals. |
| Primary node | The dominant process hypothesis: PRO, ANX, DEF, or SUP. |
| Threshold | A, B, B-to-C, or C tolerance estimate. |
| Action | The therapist move selected because of the node and threshold. |
| Safeguard | How the therapist protects safety, alliance, positives, and function. |
| Functional target | The Mini-ICF-APP related capacity or participation goal to re-check. |

# 2. Rater training sequence

1. Read the main manuscript and Supplementary Material S1, focusing on node and threshold definitions.
2. Jointly annotate 10-15 short clips or written vignettes with an expert guide; discuss disagreements aloud.
3. Independently code a calibration set of approximately 20 decision episodes.
4. Compare ratings, refine ambiguous rules, and document boundary cases before formal coding.
5. For early research, provisional targets may be node kappa >= 0.70 and threshold kappa >= 0.65; these are proposed feasibility targets, not established standards for PAD-S/CSA.

# 3. Example A: psychosis-spectrum fragility and ANX downshift

**Table S2.2. Annotated micro-sequence A: ANX thresholding in SMI.**

| **Time** | **Speaker** | **Utterance / cue** | **Episode-line coding** | **Clinical rationale** |
| --- | --- | --- | --- | --- |
| 00:00 | Therapist | When you noticed the other patient looking at you in group, what happened inside? | Trigger | Interpersonal scrutiny cue in a psychosis-spectrum context. |
| 00:06 | Patient | I felt my stomach flip. Then everything got foggy and I could not follow the room. | ANX, B-to-C | Smooth-muscle anxiety plus CPD risk; not a moment for deeper challenge. |
| 00:14 | Therapist | Let us stop there. Feet on the floor. Look around and name two things in the room. | Action: downshift; safeguard: grounding | The next move protects orientation and prevents further destabilization. |
| 00:28 | Patient | I can see the window and your notebook. It is a bit clearer now. | ANX, B | Threshold improves from B-to-C toward B; still fragile. |
| 00:36 | Therapist | Good. For next group, we plan an exit signal and a two-minute participation target. | PRO micro-step; functional target | Progress is translated into group participation with external scaffold. |

# 4. Example B: progress followed by shame collapse

**Table S2.3. Annotated micro-sequence B: SUP safeguarding after progress.**

| **Time** | **Speaker** | **Utterance / cue** | **Episode-line coding** | **Clinical rationale** |
| --- | --- | --- | --- | --- |
| 00:00 | Therapist | You kept the appointment and sent the message you planned. What do you notice as we say that? | Trigger / PRO cue | Positive functional step is named. |
| 00:08 | Patient | It was nothing. A normal person would do more. I am disgusting. | SUP, B | Progress triggers punitive self-attack; shame rises but patient remains oriented. |
| 00:16 | Therapist | Let us protect the part that did the step before that attacking voice takes it away. | Action: protect positives; de-shame | The intervention safeguards progress rather than increasing demand. |
| 00:28 | Patient | I want to push it away, but I did do it. | PRO with SUP monitoring | Agency returns; SUP remains active but less dominant. |
| 00:36 | Therapist | For this week: one self-care action, then we re-check whether the attack returns. | Functional target and re-check | Micro-progress is linked to self-care and monitoring. |

# 5. Example C: DEF-passivity sequence for supervision

**Table S2.4. Annotated micro-sequence C: DEF-passivity and graded agency.**

| **Step** | **Patient cue** | **Node / threshold** | **Therapist move** | **Why this move?** |
| --- | --- | --- | --- | --- |
| 1 | I do not know. You decide. Just tell me what I should do. | DEF, B | Could we look at this together? What would you like help with first - one small thing? | Bypass intense challenge; invite agency while keeping the task small. |
| 2 | Maybe I am waiting for you to take over. | DEF, B | I notice you are waiting for me to take over and decide. Do you see how that is happening right here between us? | Clarify here-and-now pattern without humiliating the patient. |
| 3 | If I choose, I might disappoint you. | DEF + SUP, B | Let us slow that down: one wish, then we check the fear of disappointing me. | Node switch: passivity is linked with shame/fear; keep graded format. |

# 6. Clip-level scoring template

**Table S2.5. Rater scoring sheet. Duplicate rows as needed.**

| **Clip ID** | **Dominant node** | **Peak threshold** | **Calibrated format adherence (0-3)** | **Safety adherence (0-3)** | **Functional target / notes** |
| --- | --- | --- | --- | --- | --- |
|  |  |  |  |  |  |
|  |  |  |  |  |  |
|  |  |  |  |  |  |

# 7. Safety flags requiring clinician review

- Threshold C, B-to-C drift, or emerging CPD/dissociation.
- Self-harm urges, suicidal ideation, severe shame collapse, or rapid withdrawal after progress.
- Psychotic intensification, persecutory guilt, command experiences, or marked disorganization.
- Any disagreement between patient feedback and therapist interpretation, especially when the patient reports feeling harmed, pressured, or misunderstood.
