# Supplementary material for: From symptoms to function: the PAD-S decision matrix for severe mental illness—a transdiagnostic clinical translation framework for ICD-11/ICF-aligned psychotherapy documentation
Source: Front Psychiatry. 2026 Jul 1;17:1689702. doi: 10.3389/fpsyt.2026.1689702 (PMC13370903; doi:10.3389/fpsyt.2026.1689702)
Supplement: Supplementary file 3 [file Table3.docx]

**Supplementary Material S3**

*Advanced psychodiagnostic calibration tables and phrase banks (optional clinical support)*

# Purpose and scope

This supplement preserves the more psychodynamically detailed calibration material from the earlier supplement set while making it optional and clinically transparent. It is intended for advanced supervision and training, especially when therapists need to decide whether to use standard or graded intervention formats. It is not required for understanding the main manuscript and is not a standalone diagnostic instrument.

| **How to read these tables:** The columns describe working positions on a resistance-fragility continuum. They are momentary clinical hypotheses for intervention calibration, not fixed personality labels. In SMI, the right side of the continuum requires more conservative thresholds and stronger safeguards. |
| --- |

# 1. Resistance-side calibration: organized avoidance with relatively preserved tolerance

**Table S3.1. Resistance-side PAD-S calibration support.**

| **Dimension** | **Moderate resistance** | **Moderate-high resistance** | **High resistance** | **High resistance plus repression** |
| --- | --- | --- | --- | --- |
| Observable markers | Names the problem; coherent story; anxiety mostly striated; alliance may strengthen with focus. | Oscillates between reflection and concrete detail; anxiety spikes; detours under pressure. | Intellectualizing, debating, over-control, competitive stance; anxiety remains mostly striated. | Flat prosody, restricted affect, somaticizing, pseudo-resilience, limited emotion words. |
| Typical risks | Sterile insight; no action despite understanding. | B-to-C drift if pressed too quickly; strain or rupture. | Power struggle, avoidance disguised as analysis, dropout if humiliated. | Shutdown; apparently calm compliance while affect is bypassed. |
| Preferred format | Standard format may be possible: clarify, block avoidance, deepen briefly. | Hybrid: partial clarification plus early regulation and frequent re-check. | Standard active defense work only if alliance and ANX are stable; monitor closely. | Graded format: gentle clarification, cost-linking, emotion-word building. |
| Example prompt | What do you feel toward X for 10-20 seconds? | Two sentences, then pause and notice your body. | You are analyzing; what is underneath that right now? | What is the cost of postponing this feeling again? |
| Safeguard | Link to function and re-check action. | Slow down when body signs increase. | Avoid humiliation; name the positive intent of defenses. | Do not interpret flatness as safety; check body and relational contact. |

# 2. Fragility-side calibration: reduced tolerance, CPD risk, and need for scaffolding

**Table S3.2. Fragility-side PAD-S calibration support.**

| **Dimension** | **Low fragility** | **Moderate fragility** | **High fragility** | **Severe fragility** |
| --- | --- | --- | --- | --- |
| Observable markers | Anxiety shifts toward smooth-muscle signs with intimacy; abrupt detours; fluctuating reflective capacity. | Micro-attacks on gains; withdrawal; blurring; shame after closeness or help. | Early CPD, tunnel vision, fogging, dissociation, primitive defenses, joy-to-attack-to-collapse. | CPD or disorganization at minimal load; thought blocking; psychotic intensification; minimal reflective capacity under relational load. |
| Typical risks | Rupture after help; oscillating tolerance; hidden shame. | Collapse after progress; hopelessness; disengagement. | Disorganization in groups; safety incidents; self-harm urges after positives. | Rapid decompensation; exposure/confrontation may be unsafe without external scaffold. |
| Preferred format | Graded: regulate first, then micro-PRO. | Graded only: protect positives and use very small steps. | Graded plus scaffold: seconds-long windows, orienting, team scripts. | Stabilize first; postpone deepening; external supports and crisis protocols as needed. |
| Example prompt | Name three things, then one sentence about what you need. | If that attacking voice spoke, what would it say? Let us keep this small. | Hold this for two seconds, then rest. We protect what just emerged. | Feet on floor. Where are we right now? Let us slow down. |
| Safeguard | Frequent threshold checks. | De-shame; protect positives before any challenge. | No sustained activation; plan re-check and supports. | Human-final safety review; do not code or automate beyond clinician supervision. |

# 3. SUP/shame patterns across the continuum

**Table S3.3. SUP/shame calibration patterns.**

| **Dimension** | **Organized guilt / self-criticism** | **Defended shame** | **Fragile shame** | **Severe shame / persecutory self-attack** |
| --- | --- | --- | --- | --- |
| Phenomenology | Proportionate guilt; repairable self-criticism; patient can stay engaged. | Perfectionism, cynical detachment, contempt, muted shame, avoidance of being seen. | Global defectiveness, annihilating shame, collapse when helped, praised, or seen. | Psychotic or persecutory self-attack; bizarre guilt; confusion, agitation, or self-harm risk. |
| Observable behavior | Admits fault, repairs, can reflect after clarification. | Apologizes excessively, dismisses gains, shifts topic, one-up stance, flat affect. | Withdraws, gaze aversion, shutdown, dissociation, self-harm urges after progress. | Disorganization, persecutory tone, accusatory delusions, rapid collapse under minimal relational load. |
| Immediate PAD-S response | Reality-test punitive belief; clarify cost; maintain standard dose if ANX stable. | Protect positives early; name pattern; switch to graded format when tolerance narrows. | Immediate shame repair plus regulation; postpone deepening; coordinate supports. | Safety first: orient, regulate, assess risk, use external containment and higher level of care when indicated. |
| Later work | Values-based agency and reparative action. | Flexible standards; safe assertiveness; link stance to needs. | Micro-assertions, compassion with limits, gradual interest/joy tolerance. | Long-term shame repair and multimodal care; no isolated high-intensity exposure. |

# 4. Cross-orientation phrase bank

**Table S3.4. Cross-orientation wording to reduce ISTDP-specific jargon.**

| **Clinical aim** | **Psychodynamic/EDT wording** | **CBT/skills-compatible wording** | **Supportive/rehabilitation wording** |
| --- | --- | --- | --- |
| Reduce avoidance without humiliation | Could we notice how this analysis protects you right now? | What is the thought or behavior that helps you avoid the difficult feeling? | Let us name the protective habit and choose one doable step. |
| Regulate ANX | Stay with the body signal for two seconds, then we pause. | Let us use grounding before we continue. | Feet on the floor; we will slow this down and keep it manageable. |
| Protect positives after PRO | Let us protect the part that made this step before the attack begins. | Notice the achievement and the self-critical thought as two separate events. | This step counts. We write it down and keep the next task small. |
| Work with SUP/shame | Can we look at the attacking voice without believing it? | What evidence supports and contradicts this global self-judgment? | How can we respond to that harsh voice in a way that keeps you safe today? |
