# Supplementary material for: From symptoms to function: the PAD-S decision matrix for severe mental illness—a transdiagnostic clinical translation framework for ICD-11/ICF-aligned psychotherapy documentation
Source: Front Psychiatry. 2026 Jul 1;17:1689702. doi: 10.3389/fpsyt.2026.1689702 (PMC13370903; doi:10.3389/fpsyt.2026.1689702)
Supplement: Supplementary file 4 [file Table4.docx]

**Supplementary Material S4**

*ICD-11/ICF/Mini-ICF-APP bridge, documentation templates, and implementation checklist*

# Purpose and claim calibration

This supplement supports the function-first documentation logic of the main manuscript. It shows how session-level PAD-S/CSA episode lines can be linked to ICD-11/CDDR severity language and ICF/Mini-ICF-APP functioning language. This is an implementation-oriented mapping hypothesis, not a validated ontology, formal ICD-11-ICF crosswalk, or automated recommendation system.

**Table S4.1. Three complementary levels: diagnosis, functioning, and session process.**

| **Level** | **Clinical question** | **Example output** | **What not to claim** |
| --- | --- | --- | --- |
| ICD-11/CDDR | What is the diagnostic syndrome and severity context? | Psychosis-spectrum disorder with recurrent destabilization; severe persistent depressive presentation with risk. | Do not infer session process from diagnosis alone. |
| ICF / Mini-ICF-APP | Which capacities, activities, and participation domains are restricted? | Endurance, planning, contacts, self-care, group integration, occupational capacity. | Do not treat functioning ratings as a full psychotherapy formulation. |
| PAD-S/CSA episode line | What happened in this session and how did the therapist calibrate the next move? | Trigger -> observed response -> node -> threshold -> move -> safeguard -> functional target -> re-check. | Do not automate treatment decisions from episode lines. |

# 1. Severity-to-function phrase bank

**Table S4.2. ICD-11 severity language translated into ICF/Mini-ICF-APP functioning targets.**

| **Working severity / tolerance pattern** | **Typical PAD-S read-out** | **Likely Mini-ICF-APP focus** | **Copy-ready documentation phrase** |
| --- | --- | --- | --- |
| Mild to moderate impairment; tolerance mostly A to B | High resistance or organized avoidance; coherent collaboration; PRO available. | Assertiveness, decision making, flexibility, rule adherence, work/education follow-through. | At A-to-B tolerance, expect improved assertiveness and decision making when avoidance is clarified and action is kept concrete. |
| Moderate impairment; tolerance narrows under attachment or stress | Mixed presentation; ANX B or B-to-C; SUP micro-attacks after help or progress. | Endurance, planning, dyadic relatedness, self-care regularity, treatment engagement. | Under relational stress, tolerance narrows toward B-to-C; use graded steps and re-check endurance and planning within 6-8 weeks. |
| Severe impairment; minimal load can trigger collapse | Fragile presentation; ANX/SUP B-to-C or C; CPD, shame collapse, or self-harm risk. | Endurance, group integration, self-care, occupational capacity, safety behavior, external scaffolding. | Minimal load may produce C-level collapse; prioritize safety, seconds-long exposure windows, external scaffold, and re-check functional stability within 8-12 weeks. |

# 2. Mini-ICF-APP related target examples

**Table S4.3. Example Mini-ICF-APP related targets for PAD-S/CSA documentation.**

| **Functional domain (examples)** | **PAD-S cue that may block function** | **Example target** | **Re-check interval** |
| --- | --- | --- | --- |
| Planning and structuring tasks | ANX B-to-C after complex demands; DEF passivity. | Plan one task with two steps; document whether anxiety or passivity interrupts completion. | 2-4 sessions or 6-8 weeks. |
| Flexibility | DEF rigidity or intellectualizing; SUP shame after uncertainty. | Practice one alternative response when plans change; track shame/ANX response. | 4-8 weeks. |
| Decision making | DEF passivity; fear of disappointing therapist or team. | Make one low-risk choice in session and one outside session; re-check SUP/ANX. | Next session. |
| Endurance | ANX overload, CPD, fatigue after relational load. | Participate in a brief structured activity with agreed pause/exit signal. | 2-6 weeks. |
| Contacts with others | SUP shame, DEF withdrawal, psychosis-spectrum threat sensitivity. | One graded contact with clear duration and recovery plan. | 2-4 sessions. |
| Group integration | ANX/CPD in groups; shame after being seen. | Attend one group segment with exit plan; document threshold before and after. | 1-4 weeks. |
| Self-care | SUP self-neglect or self-attack after progress. | One small self-care action after protecting positives; re-check self-attack. | Next session. |
| Occupational capacity / role participation | ANX B-to-C under performance pressure; SUP collapse after feedback. | One micro-work or role task with safeguard and debrief. | 4-12 weeks. |

# 3. Documentation templates

**Table S4.4. Documentation templates for clinical and research workflows.**

| **Use case** | **Template** | **Example** |
| --- | --- | --- |
| Routine note | Trigger -> response -> node/threshold -> move -> safeguard -> functional target -> re-check. | Manager conflict -> stomach flip/fogging -> ANX B-to-C -> grounding and postponing challenge -> orienting -> endurance/planning target -> next session. |
| Supervision | What cue did I respond to? What node did I assume? What threshold did I miss? What would be the safer next move? | I interpreted passivity as DEF but missed SUP fear of disappointing me; next time use graded agency plus shame check. |
| Team handoff | Diagnosis/severity + functional domain + session process risk + agreed safeguard. | Psychosis-spectrum fragility; group integration impaired; CPD under scrutiny; use exit plan and two-minute participation target. |
| ROM/MBC action rule | If outcome worsens or function stalls, inspect recent episode lines for ANX/SUP escalation or insufficient safeguards. | Drop in self-care after progress suggests SUP backlash; protect positives before adding tasks. |
| Transcript annotation study | Segment, label node/threshold, record action and safeguard, then adjudicate disagreements. | Raters disagree DEF vs SUP; consensus notes passivity driven by shame fear, so SUP secondary. |

# 4. Implementation checklist

**Table S4.5. Minimum implementation checklist for SMI services.**

| **Before implementation** | **Minimum requirement** |
| --- | --- |
| Training | Therapists/rater trainees can distinguish node, threshold, action, and safeguard in short examples. |
| Clinical governance | Local crisis protocols, informed consent, privacy rules, and documentation responsibilities are explicit. |
| Human-final review | All annotations and summaries are reviewed by a clinician before use in care, supervision, or benchmarking. |
| Burden check | Episode-line documentation can be completed without disrupting therapy or adding excessive administrative load. |
| Equity and accessibility | Digital use considers SMI-specific needs, digital health literacy, cognitive load, and supported access. |
| Evaluation plan | Reliability, feasibility, acceptability, safety, functional outcomes, and implementation burden are measured. |

# 5. Optional computational workflow details

Technical teams may use the episode-line schema to build annotation datasets, supervision dashboards, or process-research pipelines. These tools should remain downstream of clinician-adjudicated labels. Suitable early tasks include reliability estimation, segmentation feasibility, error analysis of AI-generated summaries, and association between safeguard use and functional outcomes. Unsuitable early tasks include automated treatment selection, unsupervised risk decisions, or substituting generated text for clinician review.
