# Supplementary figures and images for: From symptoms to function: the PAD-S decision matrix for severe mental illness—a transdiagnostic clinical translation framework for ICD-11/ICF-aligned psychotherapy documentation
Source: Front Psychiatry. 2026 Jul 1;17:1689702. doi: 10.3389/fpsyt.2026.1689702 (PMC13370903; doi:10.3389/fpsyt.2026.1689702)

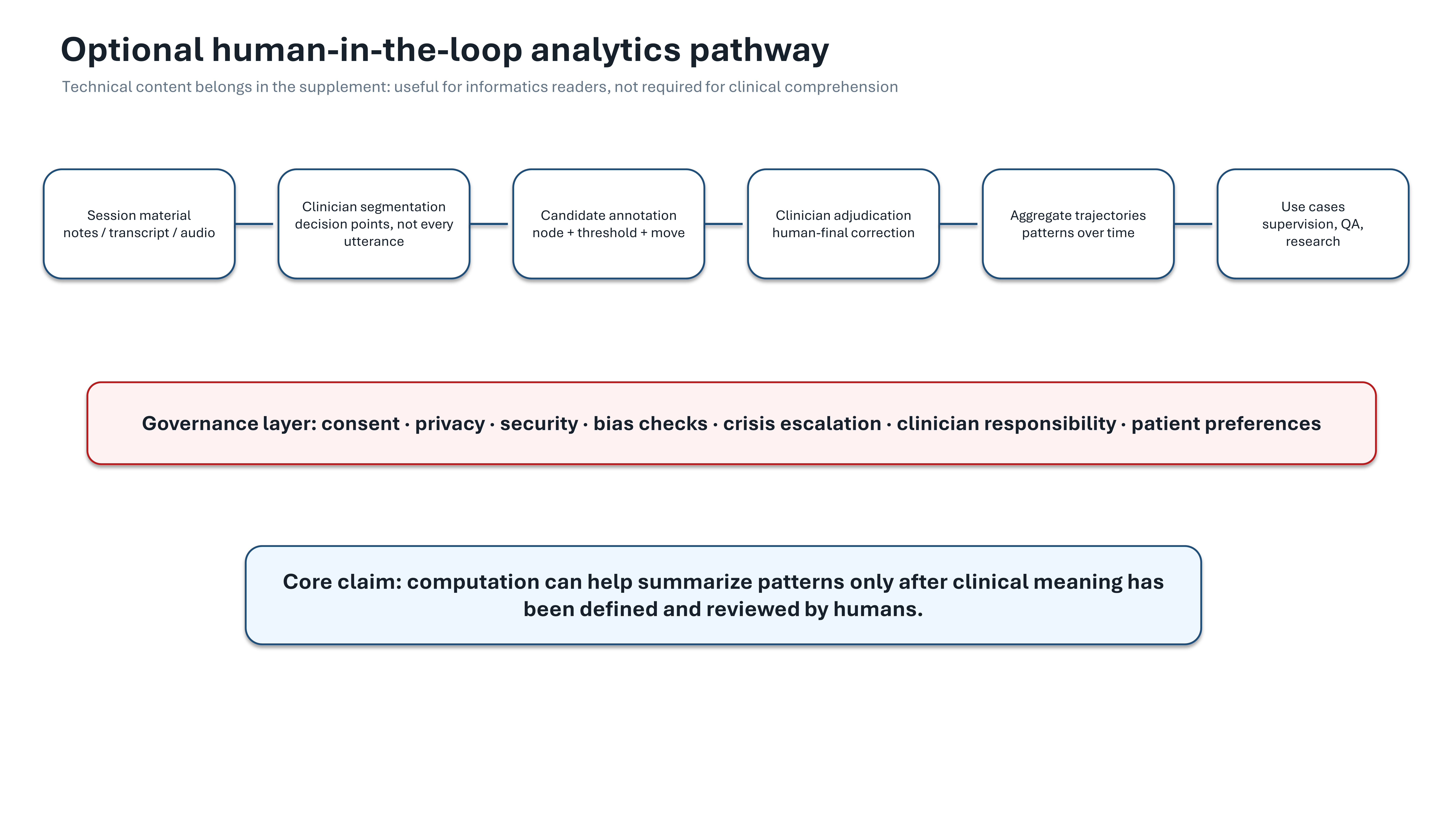

Supplement: Supplementary file 5 [file Image1.tif]
